# Progress and prospects in metabolic engineering approaches for isoprenoid biosynthesis in microalgae

**DOI:** 10.1186/s13068-025-02665-y

**Published:** 2025-06-18

**Authors:** Sonia Mohamadnia, Borja Valverde-Pérez, Omid Tavakoli, Irini Angelidaki

**Affiliations:** 1 Department of Chemical and Biochemical Engineering, Søltofts Plads 228A, Technical University of Denmark, DTU, 2800 Lyngby, Denmark; 2 Department of Environmental and Resource Engineering, Technical University of Denmark, Bygningstorvet 115, 2800 Lyngby, Denmark; 3 School of Chemical Engineering, College of Engineering, University of Tehran, Tehran, 14176 Iran

**Keywords:** Isoprenoid, Microalgae, Metabolic engineering, Carotenoids

## Abstract

Isoprenoids constitute a large and various number of bio-compounds, with many profitable applications in pharmaceutical, nutraceutical, and industrial fields. The complexity of isoprenoid molecules leads to a challenging, expensive, and environmentally unfriendly chemical synthesis of these metabolites. In addition, the awareness and desire of many consumers for products generated by natural microbial processes has increased recently. Metabolic engineering tools and synthetic biology strategies have been used as a means for the enhancement and optimization of the natural isoprenoid biosynthetic pathways of wild strains. Microalgae as production organisms have been manipulated for the bioproduction of diverse isoprenoids. Particularly when cultivated in unsuitable conditions (such as wastewater, unbalanced nutritional sources, and distinct environmental conditions), microalgae can adjust their metabolic pathways and generate compounds with significant technological potential. Several metabolic engineering approaches have been developed, modifying the metabolic pathways in microalgae to redirect the flow of carbon toward isoprenoid biosynthesis, including pathway engineering, strain improvement, and synthetic biology. In this review, some beneficial features of these high-value metabolites are summarized. Besides, recent advancements in metabolic engineering approaches for the biosynthesis of isoprenoids are discussed in detail. At last, the viewpoints and challenges for the biosynthesis of novel compositions with isoprene units in the microalgae are also included.

## Introduction

Isoprenoids (terpenes/terpenoids) are one of the most abundant and structurally highly complex natural products, which are utilized in various industries such as pharmaceutical, medical, nutraceutical, agricultural, fragrance, rubber, and advanced biofuels [78, 129]. Isoprenoids are hydrocarbons made up of two or more isoprene units, each consisting of five carbon atoms organized in a special pattern [13]. Several reactions, e.g., oxidation, reduction, rearrangements, cleavage of the rings, or cyclization, lead to the conversion of isoprenoid structures to cyclic or acyclic structures [29]. Hence, they are categorized based on the number of isoprene units appearing in their composition (e.g., hemiterpenoids (1 unit), monoterpenoids (2 unit), sesquiterpenoids (3 units), diterpenoids (4 units), sesterterpenoids (5 units), triterpenoids (6 units), tetraterpenoids (8 units), and polyterpenoids (> 8 units); [43]. In the configuration of all isoprenoid structures, isopentenyl pyrophosphate (IPP) and its isomer dimethylallyl pyrophosphate (DMAPP) are the two key precursor molecules [14, 27, 128]. The 2-C-methyl-d-erythritol-4-phosphate (MEP) pathway, in bacteria and plastids of plant cells, and the mevalonate (MVA) pathway, in most eukaryotes, archaea, and certain bacteria, are responsible for the production of IPP and DMAPP [27]. In addition, IPP and DMAPP come from acetyl-CoA or pyruvate/glyceraldehyde-3-phosphate in the MVA or MEP pathway, respectively [146]. Currently, plants, bacteria, and animal tissues are the major resources in the production of terpenoids for industrial applications [15, 82, 89]. Previous publications have dealt with cultivation, harvesting, recovery, purification, extraction, and conversion, and have the ultimate intention to develop an economically feasible commercial process. Nevertheless, the high costs associated with the process and low overall productivity make the industrial terpenoids production from microbial sources unsuitable. As a way to address the limitations of unspecific isoprenoid production, which limits the specific yields of isoprenoids, metabolic engineering approaches have been applied as an efficient way to synthesize isoprenoids from microbial processes [58, 89]. Among microbial sources, microalgae have several clear benefits for isoprenoid production via metabolic engineering approaches over conventional microbial hosts such as *Escherichia coli* and *Saccharomyces cerevisiae*. First, a large number of microalgae have native MVA or MEP pathways and naturally create a variety of isoprenoids, which reduces the requirement for complex heterologous pathway construction [80]. Additionally, they can fix atmospheric CO₂ through photosynthesis, which minimizes the need for costly organic feedstocks and offers a sustainable, carbon–neutral pathway to isoprenoid biosynthesis [49]. Furthermore, subcellular compartmentalization in microalgae, as present in chloroplasts, allows pathways to be physically separated and allows for focused metabolic engineering to achieve higher yields of target compounds [133]. In addition, advancements in genetic tools, including Clustered Regularly Interspaced Short Palindromic Repeats (CRISPR) or CRISPR-associated (Cas) systems and organelle-specific promoters, have improved the metabolic flexibility and engineering potential of microalgae [30]. Microalgae also facilitate the co-production of isoprenoids with other valuable bioproducts, such as proteins and lipids; hence, promoting integrated biorefinery methods [95, 130]. Besides, as compared to traditional microorganisms as hosts, microalgae cultivation in selected conditions, like high salinity or light-dependent systems, lowers the risk of contamination. Finally, microalgae are a resource-efficient and environmentally friendly platform for industrial isoprenoid synthesis since they can be cultivated on non-arable land with wastewater and less freshwater [80, 86].

**Fig. 1:**
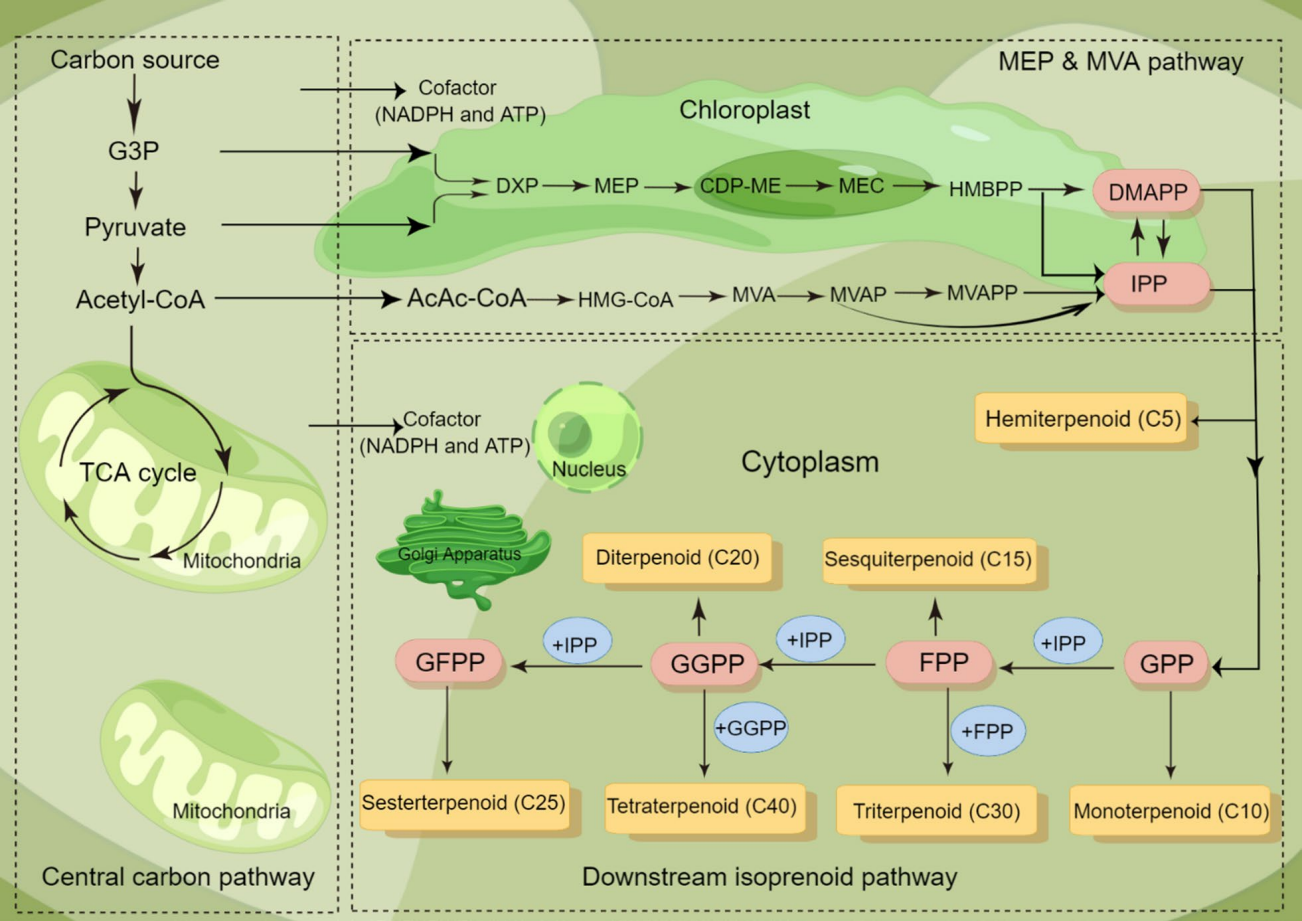
The metabolic pathway of isoprenoid biosynthesis is divided into three distinct parts. The carbon flux to the isoprenoids pathway supplies the necessary precursors and cofactors for the MVA/MEP pathways, which then produce the common precursors DMAPP and IPP. These two precursors are then condensed to generate GPP, FPP, GGPP, and GFPP, which are catalyzed to form various isoprenoids. 2-C-methyl-d-erythritol-4-phosphate (MEP), mevalonate (MVA). Isopentenyl PyroPhosphate (IPP). DiMethylallyl PyroPhosphate (DMAPP). MeVAlonate-5-diphosphate (MVAPP). MeVAlonate-5-Phosphate (MVAP). 3-Hydroxy-3-MethylGlutaryl-CoA (HMG-CoA). AcetoAcetyl-CoA (AcAcCoA). (E)−4-Hydroxy-3-Methyl-But-2-enyl diphosphate (HMBPP). 2-C-Methyl-D-Erythritol 2,4-cyclodiphosphate (ME-cPP). 4-(cytidine-5ʹ-diphospho)−2-C-methyl-D-Erythritol (CDP-ME). 1-Deoxy-d Xylulose 5-Phosphate (DXP). Glyceraldehyde-3-Phosphate (G3P). Geranyl diphosphate (GPP). Farnesyl diphosphate (FPP). GeranylGeranyl diphosphate (GGPP). GeranylFarnesyl diphosphate (GFPP)

Table 1 presents numerous publications on microalgal bioprocess and biorefinery for sustainable isoprenoid synthesis at lower production costs, which have been conducted mainly in *Chlamydomonas reinhardtii* (*C. reinhardtii*) and *Phaeodactylum tricornutum* (*P. tricornutum*). Photosynthesis of microalgae requires the presence of chlorophyll and carotenoid pigments, which are derived from isoprenoid metabolism. Therefore, to ensure effective photosynthesis, a directed carbon flux towards the isoprenoid pathway in microalgae is needed. Isoprenoids synthesis pathway in microalgae is subdivided into three steps (Fig. 1). At the first stage, precursors and cofactors, e.g., glyceraldehyde-3-phosphate (G3P), pyruvate, acetyl-CoA, NADPH, and ATP, are produced either through glycolysis pathway (organic carbon sources) or Calvin cycle (inorganic carbon sources). In the second step, IPP and DMAPP are derived from MEP and MVA pathways and are sequentially condensed to each other through isoprenyl diphosphate synthases [27]. It is worth noticing that diatoms maintain both MEP and MVA pathways for the generation of the precursors, unlike other algae species that contain only one of the biosynthetic pathways (MEP and MVA) [7, 85]. For example, most green algae only maintain the MEP pathway [47, 100, 113]. Among red algae, the MVA pathway has been absent in the lineages of *Cyanidioschyzon*, *Porphyridium*, and *Chondrus*, but is present in *Galdieria*. In the third step, different kinds of prenyl diphosphates, including geranyl diphosphate (GPP), farnesyl diphosphate (FPP), geranylgeranyl diphosphate (GGPP), and geranylfarnesyl diphosphate (GFPP), are synthesized [78]. These are the trigger molecules for the hemiterpenes (C5), monoterpenes (C10), sesquiterpenes (C15), diterpenes (C20), sesterterpenes (C25), triterpenes (C30), and tetraterpenes (C40) [67]. Actually, this stage involves the linear condensation of isoprene units to produce prenyl diphosphates with varying chain lengths. Typically, prenyltransferases catalyze these reactions and lead to a wide range of complicated compounds that have been addressed [63, 69]. The cytochrome P450 superfamily of enzymes (P450s or CYPs) is involved in the subsequent modification steps, such as oxidation and hydroxylation, of these terpenoid compounds. Ultimately, the prenyl diphosphate molecules enter different pathways and turn out several products such as sterols, alkaloids, carotenoids, etc. [82].

**Table 1:** Summary of applications of isoprenoids derived from microalgae

Type of isoprenoids	Chemical formula	Properties/applications	Strain	References
Monoterpene (C10)				
Limonene	C_10_H_16_	Biofuels, flavoring agent, anticancer, antiobesity	*Chlamydomonas reinhardtii, Synechococcus elongatus, Anabaena, Synechocystis*	[98, 121]
Pinene		Biofuels	*Synechococcus *sp*.*	[139]
β-myrcene		Essential oils, flavoring, and cosmetics	*Ochtodes secundiramea*	[108]
Geraniol	C_10_H_18_O	Insect repellent, additive in the food and beverage industries to enhance the flavors	*Phaeodactylum tricornutum*	[35]
Linalool		Antimicrobial agent, aroma compound	*Chlorella* sp.*, Chlamydomonas* sp., *Oocystis pusilla*	[46]
Menthol	C_10_H_20_O	Flavor agent	*Phormidium autumnale, Chlorella vulgaris*	[36, 126]
Sesquiterpene (C15)				
β-bisabolene	C_15_H_24_	Biofuels	*Chlamydomonas reinhardtii*	[122]
β-caryophyllene		Anti-inflammatory, antimicrobial,	*Synechocystis*	[112]
Patchoulol	C_15_H_26_O	Anti-inflammatory	*Chlamydomonas reinhardtii*	[73]
Farnesol		Flavor and fragrance agents, chemopreventive and anticancerous	*Botryococcus braunii*	[102]
Santalol	C_15_H_24_O	Fragrances, antitumor, anticancer, biofuels	*Chlorella* sp*.*	[9, 129, 134]
Domoic acid	C_15_H_21_NO_6_	Neurotoxin	*Pseudo-nitzschia*	[12]
Abscisic acid	C_15_H_20_O_4_	Growth hormone	*Nannochloropsis oceanica*	[87]
Diterpene (C20)				
Taxadiene	C_20_H_32_	Anticancer	*Chlamydomonas reinhardtii*	[74]
Gibberellic acid	C_19_H_22_O_6_	Growth hormone, supplement elements in the human diet, and drugs in disease treatment	*Scenedesmus obliquus, Chlorella* sp*., Chlorella sorkiniana*	[21, 120]
Sesterterpenes (C25)				
Haslenes	C_25_H_40_	Anticancer	*Haslea ostrearia*	[93, 127]
Triterpene (C30)				
Betulin	C_30_H_50_O_2_	Anticancer, HIV treatment	*Phaeodactylum tricornutumPhaeodactylum tricornutum,*	[25]
Lupeol	C_30_H_50_O	Anticancer and used in treatment of HIV		
Botryococcene	C_34_H_58_	Biofuel	*Botryococcus braunii, Chlamydomonas reinhardtii*	[59, 101]
Squalene	C_30_H_50_	Anti-inflammatory, vaccine adjuvant, antioxidant, antiaging compound		
Phytosterol	C_29_H_50_O	Diverse health and pharmaceutical properties	*Chlamydomonas reinhardtii*	[22]
Ergosterol	C_28_H_44_O			
Tetraterpene (C40)				
β-carotene	C_40_H_56_	Antioxidant, anticancer, antiinflammatory	*Dunaliella salina, Chlamydomonas reinhardtii, Scenedesmus* sp. CPC2	[17, 64, 141]
Lycopene		Antioxidants are used as treatments for cardiovascular diseases and prostate cancer	*Botryococcus braunii*	[53, 101]
Fucoxanthin	C_42_H_58_O_6_	Antiobesity, anticancer, antioxidant	*Phaeodactylum tricornutumPhaeodactylum tricornutum, Tisochrysis lutea*	[96]
Astaxanthin	C_40_H_52_O_4_	Antioxidant, UV protection, food colorant, antiaging, immune enhancement, pharmaceutical, antihypertensive, and anticancer, anti-inflammatory	*Haematococcus pluvialis, Chlorella zofingiensis, Dunaliella viridis*	[83, 84, 104]
Canthaxanthin	C_40_H_52_O_2_	Food coloring agent	*Dunaliella viridis*	[83]
Neoxanthin	C_40_H_56_O_4_	Anticancer	*Chlamydomonas reinhardtii*	[60, 80, 129]
Violaxanthin		Anti-inflammatory		
Lutein	C_40_H_56_O_2_	Antioxidant, prevention serious eye disease		
Zeaxanthin		Prevention serious eye disease

**Fig. 2:**
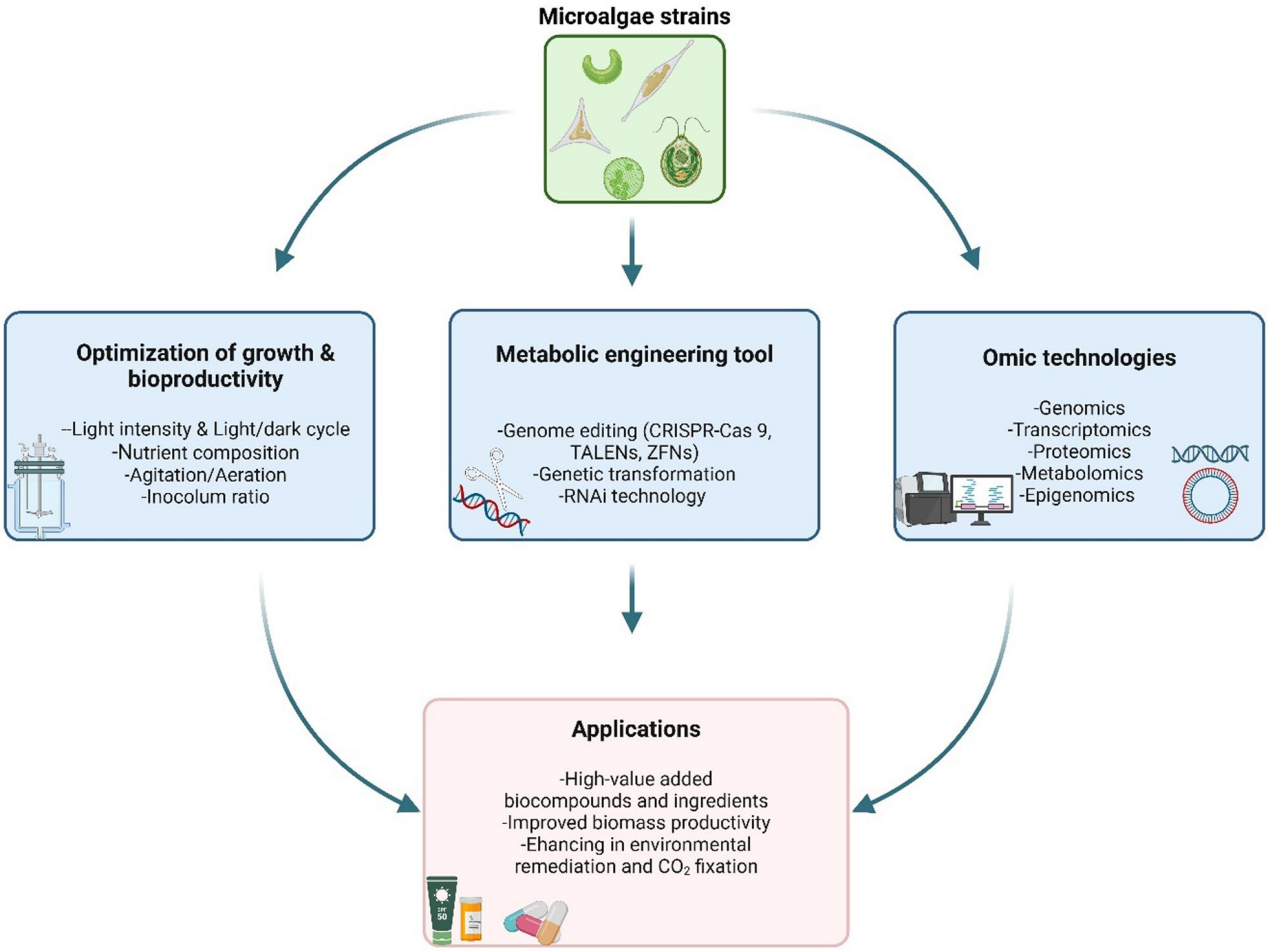
An overview of microalgae strain development using various tools/approaches

There are many studies to promote the production of carotenoids, phytosterols, triacyglycerol, and provitamin A over MVA and/or MEP ways via genome modification in plants [66]. Perpetuated structures, species-dependent molecules, and prominent metabolic and physiological characteristics, proposed microalgae as an excellent origin of isoprenoids among all natural sources [7]. Nowadays, growing information and data availability from genomic and transcriptomic studies the isoprenoids biosynthesis opens for possibility for genetic manipulation of microalgal metabolism [62]. Figure 2 presents the current state of the art concerning the approaches for promoting and enhancing the bioproduction of high-value metabolites in microalgae, including natural diversity, growth, and productivity optimization combined with omics technologies and genetic engineering tools, with a focus on application in a variety of industrial sectors.

The main strategy is supplying more precursors, mainly IPP and DMAPP, through metabolic engineering strategies.

The main five strategies that have been extracted from the studies and experiments to overcome precursor limitations are [16, 48, 146]:

1. Overproduction of the enzymes in the rate-limited reactions in the biosynthetic pathway of isoprenoids.
2. Knockout of some deficient genes and regulators in the biosynthetic pathway.
3. Precursor production by engineered host cells through new metabolic pathways.
4. Adequate presence of cofactors in biosynthetic routes.
5. Diminishing the rate of reactions that lead to the production of unfavorable metabolites, thus increasing the production of desirable products and essential intermediates from the metabolic pathways.

Overcoming the availability of precursors for the wide range production of isoprenoids is one of the major issues facing researchers. On the other hand, formation of certain isoprenoids (species-dependent) in commercial scale in a heterogeneous host is also of great importance.

This review initially describes some applications of well-known isoprenoids in food, energy, pharmaceuticals, and cosmetics industries. Then, the metabolic engineering strategies progression to produce isoprenoids through the biosynthetic pathway of microalgae is provided. To overcome the limitations in the sustainable production of microalgae-associated isoprenoids, enormous efforts and techniques have been developed to enhance their commercial-level production, which has also been discussed. In the last section, some of the challenges and the potential developments for advancing the production of isoprenoids are identified.

## Isoprenoids: healthful and therapeutic biocompounds

Isoprenoids or terpenoids have several important roles as protective agents, accessory and photoprotective pigments, toxins (neurotoxin), and sterols (Table 1) [7, 19, 145]. In addition there are some highly branched isoprenoid (HBI) that have several health effect such as anticancer, anti-HIV, and cytostatic [7, 93]. However, the production of terpenoids that have medical and health properties is very low. The majority of terpene compounds with health properties are nowadays taken directly from plants. However, the amount of terpene compounds in plants is modest, and their regulating mechanism is unknown [37]. Furthermore, there are many challenges with the extraction and purification of these valuable substances. Thus, there is an emerging need for biotechnological production of these valuable compounds from microbial cell factories. A new technology based on bacteria or yeasts as hosts for non-native production of isoprenoid metabolites is under development [74]. In addition, microalgae can be another suitable host for sustainable heterologous production of isoprenoids due to their easy cultivation by using light and CO_2_ as energy and carbon source [20, 74]. However, inadequate knowledge of the genomes of algae has led to slow improvement of the biosynthesis processes by genetic engineering methods (e.g., *Chlamydomonas reinhardtii* or *Phaeodactylum tricornutum* [74]. The green alga *Botryococcus braunii* synthesizes some triterpenes, e.g., botryococcene and squalene oils, that are used in the petrochemical industry [101, 131]. According to heterologous gene expression in microalgae from various organisms, e.g., plants, animals, fungi, or bacteria [3], and with respect to this point that most of the microalgae have not been chemically specified, novel isoprenoid structures may emerge through future studies. In the following sections, some of the fundamental isoprenoids derived from microalgae and their applications are introduced.

### Diterpenes

These compounds are consisted from two terpene parts, comprises of 20 carbon atoms and is one of the largest category of natural compounds with more than 18,000 products [114]. This group of isoprenoids have several structures with diverse bioactivities. Generally, diterpenoids may contain linear, monocyclic, bicyclic, tricyclic, or tetracyclic molecules [8]. They are produced from plants, animals, microalgae, some diatoms, and fungi through the mevalonate pathway, with geranylgeranyl pyrophosphate as precursor. Important diterpenoids are retinol, retinal, and phytol, which have antimicrobial and anti-inflammatory [11]. Taxadiene, ingenol-3-angelate, and forskohlin are also valuable diterpenoids with medicinal or nutraceutical properties [4, 74, 106]. Forskolin, the anticancer and antiobesity metabolite, is derived from 13R(+) manoyl oxide. Lauersen et al. [74] over-expressed diterpene synthases (diTPSs) and enzymes associated with the MEP pathway and produced heterologous diterpenoids, casbene, taxadiene, and 13R(+) manoyl oxide from the model microalgae *Chlamydomonas reinhardtii*. Taxadiene is the first committed intermediate in the synthesis of paclitaxel (Taxol). Amination of the natural tetracyclic diterpenes leads to the formation of diterpenoid alkaloids (DAs), which have complex structural properties with many stereocenters [114]. DAs form a vast group of natural metabolites with remarkably complicated structures and biological activities, which are also notoriously toxic. They have several pharmacological properties, including analgesic, antiarrhythmic, anti-inflammatory, local anesthetic, anti-arrhythmic, muscle relaxant, hypotensive, antimicrobial, antitumor, spasmolytic, psychotropic, and neurotropic [114, 135]. Although microalgae have great potential for the production of geranylgeranyl pyrophosphate (GGPP), a common precursor of pigments and diterpenoids, the production of heterologous diterpenoids in eukaryotic microalgae is still in its beginning stages.

### Sesterterpenes

Sesterterpenes (C25) which contain 25 carbon atoms are the rare found compounds among terpene. Geranylfarnesyl diphosphate (GFPP) is the linear precursor for the polycyclic structures of sesterterpenoids. These compounds have several biological activities such as anticancer, anti-inflammatory, antiprotozoal, antitubercular, antimicrobial, and modulation of receptor signaling [93].

Sesterterpenoids can be extracted from variety of natural resources including fungi, bacteria, lichens, higher plants, insects, various marine invertebrates, especially sponges, and some diatoms [77, 93]. Massé et al. [93] reported that the diatom *Rhizosolenia setigera* produces highly branched isoprenoid (HBI) sesterterpenes (haslenes). In addition, Rowland et al. [110] manifested in vitro anticancer activity of the haslenes obtained from the diatom *Haslea ostrearia*.

### Triterpenes

The structure of triterpenoids (C30) comprises of 30 carbon atoms, which make up six isoprenoid units. Over 100 triterpenoid compositions have been recognized until now, including α-amyrin, β-amyrin, dammarene-diol, and lupeol [25]. These are the precursors for the compounds used for anticancer or HIV treatment. Several metabolites under the triterpenes family can be synthesized through primary or secondary metabolism. Sterols, as the only primary substance, play an important role in adjusting the cell membrane dynamics. In addition, sterols produce secondary compounds such as steroid hormones that control human growth and development [22]. Other secondary triterpenes are phytosterols and ergosterol, applied for decreasing the blood low-density lipoprotein (LDL) cholesterol and producing vitamin D_2_ (ergocalciferol), respectively [45, 55]. Squalene, high-value added compound with numerous applications in pharmaceutical, medical, cosmetic, and food industries, convert to (*S*)−2,3-epoxysqualene, which is the precursor in triterpenoids biosynthesis [22]. Lupette et al. [90] identified brassicasterol, structurally same as cholesterol, as an element of lipid droplets (LDs) surface in nitrogen starved *Phaeodactylum tricornotum*. Other than free sterols, there are several microalga and diatom species that synthesize conjugated types of these compounds. For instance, *Thalassiosira pseudonana*, *Chaetoceros calcitrans*, and *Haslea ostrearia* contain esterified form of sterols, glycoconjugated (steryl glucosides and acylated steryl glucosides), and 23,23-dimethylcholest-5-en-3β-ol, respectively [7]. Ergosterol, usually produced in fungi, is observed in *P. tricornotum* sterol biosynthetic pathway [36]. *Cylindrotheca fusiformis* and *Nitzschia closterium* contain cholesterol that is generated by animals in most cases [109].

### Tetraterpenes (carotenoids)

Essential roles of carotenoids, composed of 40 carbon atoms (C_40_), in human life attract the attention of researchers for discovering the new strategies in their biosynthesis. Eight isoprene units are the building block of these pigments [99, 142]. Carotenoids are divided into (a) carotenes (containing carbon and hydrogen) and (b) xanthophylls (oxygenated derivatives of carotene). Color and antioxidant properties of carotenoids are owing to the conjugated double bonds in their structures. These compounds constitute a vast category of tetraterpenoids that contain several applications in industrial fields [96] such as anticancer, antioxidant, antiangiogenic, antidiabetic, antiobese, anti-inflammatory, etc. Carotenes, e.g. lycopene and β-carotene, are applied as food additive in food industry and play an important role as an antioxidant, antimalarial, and anticancer agents [42, 54, 140]. Fucoxanthin is another tetraterpenoids that plays as an accessory light harvesting xanthophyll, originated from β-carotene and transfer the light energy to the photosynthetic system [70]. Fucoxanthinol, deacetylated form of fucoxanthin, has manifested great potential in the prevention and treatment of many types of cancer [92]. Regardless of its diverse applications, the extraction of fucoxanthin from its natural resources such as alga, specially macroalga, is low and its chemical production is troublesome [38]. Manfellotto et al. [91] by exploitation genetic transformation enhanced the expression of the genes attends in the carotenoid biosynthetic pathway of *P. tricornotum*. Some of the other well-known tetraterpenes are astaxanthin, diatoxanthin, and diadinoxanthin that can be extracted from microalgae and have many biological activities [96, 111].

## Metabolic engineering approaches enhance the biosynthesis of isoprenoids

Reconstruction and optimization of the metabolic pathways to enhance the bio-production of the isoprenoid molecules through metabolic engineering and synthetic biology approaches has attracted the attention of many researchers in recent years. Even though the majority of studies has centered around *C. reinhardtii* and *P. tricornutum*, other microalgae such as *H. pluvialis*, *D. salina*, *Nannochloropsis oceanica*, *Chlorella vulgaris*, *Scenedesmus* sp., *Thalassiosira pseudonana*, and *Porphyridium purpureum* have also been explored due to their economic value [60]. The planet is host to approximately ten million algal species, representing an abundant potential bioengineering asset. However, taking advantage of these will likely necessitate further knowledge of their genetics combined with the latest advances in omic approaches such as transcriptomics, proteomics, metabolomics, and phonemics to select the desired gene. Recent reviews provide an all-inclusive look at these resources, with information on the species, mutants, omics datasets, and genetic tools at our disposal [72, 118]. Despite this, low levels of isoprenoid production in microalgae are the principal impediments currently preventing commercial development. Nevertheless, terpene synthases have been successfully expressed heterologously in microalgae, indicating their potential as a production platform.

Currently, methods to augment isoprenoid engineering are mainly limited to overexpressing rate-reducing enzymes. So, the technical progressions in gene editing tools and novel design tactics are imperative. Table 2 provides a summary of the metabolic engineering investigations for the production of isoprenoids in microalgae that have been reported in the last five years.

**Table 2:** Metabolic engineering efforts for the production of isoprenoids

Isoprenoids (class of terpenoids)	Strain	Metabolic engineering approach	Result	References
Geraniol (Monoterpenes (C10))	*P. tricornutum*	The expression of CrGES was carried out through episomal means, utilizing either the inducible alkaline phosphatase gene promoter (AP 1p) or the endogenous constitutive Phatr3_J492020p promoter	The inducible promoter (AP 1p) yielded a geraniol production of 48.60 μg L^−1^ within 72 h	[35]
Limonene (Monoterpenes (C10))	*C. reinhardtii*	Novel isopentenol utilization pathway (IUP) was introduced into the chloroplast of *Chlamydomonas reinhardtii*	The results showed 8.6-fold rise in IPP production when compared to the wild type, and a notable 23-fold increase in the production of limonene	[144]
(E)- α-bisabolene (Sesquiterpenes (C15))	*C. reinhardtii*	Overexpression of AgBs (three times) and amiRNA knock-down of SQS, AGPase, GGPPS, and PFT	The concentration of (E)-α-bisabolene was measured at 11.0 mg L^−1^ under light/dark cycles as well as mixotrophic condition	[132]
Patchoulol (Sesquiterpenes (C15))	*C. reinhardtii*	β-carotene ketolase (BKT) was expressed to perturb carotenoid biosynthesis and the host squalene synthase (SQS) was knocked down to reduce metabolic pull on FPP	The modifications resulted eightfold increase in patchoulol production using a cytosolic localized patchoulol synthase	[50]
Squalene (Sesquiterpenes (C15))	*Schizochytrium *sp*.*	Co-overexpression of SQS and HMGR gene in the recombinant strain of *Schizochytrium* sp.	The biomass and squalene titer of the recombinant strain were increased by 13.6% ± 1.2% and 88.8% ± 5.3%, respectively	[138]
Labdane diterpenes (Diterpenes (C20))	*C. reinhardtii*	Upregulation of *Cistus creticus* copal-8-ol diphosphate synthase (CcCLS)	Four labdanetype diterpene compounds (entmanoyl oxide, sclareol, labda-13-ene-8α,15-diol, and ent-13-epimanoyl oxide) were synthesized	[105]
Sclareo (Diterpenes (C20))	*C. reinhardtii*	Manipulating the 1-Deoxy-d-xylulose 5-phosphate synthase (DXS), which is the key rate-limiting enzyme of the MEP pathway, as well as overexpression of diterpene synthase fusion proteins	Up to 656 mg L^−1^ with a maximum production of 78 mg L^−1^ in a 2.5 L photobioreactor	[34]
13R(+) manoyl oxide (Diterpenes (C20))	*C. reinhardtii*	Overexpression of CfTPS2 three times, single expression of CfTPS3, and single expression of ERG20 (F96C). Moreover, the overexpression of CfTPS2, CfTPS3, and CfCYP76AH16 can lead to the production of 9-OH 13R(+) manoyl oxide	The production of 13R(+) manoyl oxide at a concentration of 40 mg L^−1^ was observed under 16:8 light/dark cycling, as well as CO_2_ exposure	[74]
Lupenol, botulin (Triterpenes (C30))	*P. tricornutum*	Simultaneous expression of *Lotus japonicus* lupeol synthase (LjLUS), *Medicago truncatula* CYP716A12 (MtCYP716A12), and *M. truncatula* P450-NADPH reductase (MtCPR) is observed	The LjLUS expressing strain was able to produce 0.1 mg L^−1^ of lupeol within a span of two days. Additionally, the engineered strain consisting of LjLUS, MtCYP716A12, and MtCPR successfully produced betulin in a pilot-scale photobioreactor with a capacity of 550-L	[25]
Astaxanthin (Tetraterpenes (C40))	*C. reinhardtii*	The modified endogenous *Chlamydomonas reinhardtii* β -carotene ketolase (CrBKT) was overexpressed through the removal of its 116 amino acid C-terminal extension which is not present in BKT homologs from other organisms	Under mixotrophic conditions, the UVM4 strain overexpressing the engineered CrBKT gene yielded 2.6–3.1 mg of astaxanthin per liter per day and 3.7–4.3 mg of ketocarotenoid per liter per day	[107]
	*C. reinhardtii*	CRISPR/Cas9-Mediated Knockout of the Lycopene ε-Cyclase	Increased astaxanthin by 2.3-fold	[103]
Lutein and β-carotene (Tetraterpenes (C40))	*C. reinhardtii*	Upregulation of DXS, DXR, and orange protein (OR) (DnaJ-like chaperone)	Overexpressing OR has been found to induce a significant increase in lutein and β-carotene titers, with respective fold increases of 1.5 and 1.3	[97]
α-carotene, lutein, β-carotene, and violaxanthin (Tetraterpenes (C40))	*C. reinhardtii*	Elevation the expression levels of both wild type *C. reinhardtii* regulatory protein ORANGE (CrOR^WT^) and the mutated (CrOR^His^) with a histidine substitution	Overexpression of CrOR^WT^ led to significant increases in the α-carotene (1.9-fold), lutein (twofold), β-carotene (2.1-fold), and violaxanthin (2.1-fold) contents in the target sample Overexpressing CrOR^His^ led to significant increases in α-carotene, lutein, β-carotene, and violaxanthin contents by 4-, 3.1-, 3.2-, and 3.6-fold, respectively	[141]
Zeaxanthin (C40)	*C. reinhardtii*	DNA-free CRISPR/Cas9, knock-out mutant-Targeting zeaxanthin epoxidase (ZEP)	Increased zeaxanthin by 56-fold	[1]
Astaxanthin (C40)	*Chlorella zofngiensis*	Overexpression of phytoene desaturase (PDS)	Increased carotenoid and astaxanthin by 32.1% and 54.1%, respectively	
Astaxanthin and canthaxanthin (Tetraterpenes (C40))	*Dunaliella viridis*	Overexpression of β-carotene hydroxylase (CRTR-B) and BKT from the chloroplast genome of *H. pluvialis*	Under high light conditions, the levels of astaxanthin and canthaxanthin were found to be 77.5 ± 7.7 μg g^−1^ DCW and 50.1 ± 0.8 μg g^−1^ DCW, respectively	[83]
Fucoxanthin (Tetraterpenes (C40))	*P. tricornutum*	Overexpression of the endogenous enzymes violaxanthin de-epoxidase (VDE), VDE-related (VDR), and Zeaxanthin epoxidase (ZEP)	The content of fucoxanthin experienced a four-fold increase	[91]
		Overexpression of heat shock transcription factor (*PtHSF1*) by nitrogen and phosphorus deficiency and upregulating the key enzyme genes, (DXS) and glycerol-2-phosphate acyltransferase 3 (GPAT3), involved in the fucoxanthin biosynthesis pathways	The content of fucoxanthin experienced a two-fold increase rather than wild type from (3 to 6 mg g^−1^ DCW)	[117]

### Precursors availability

The native glycolysis pathway in microbial hosts generates the essential precursors for isoprenoid production, namely G3P, pyruvate, and acetyl-CoA (Fig. 1). To ensure sufficient precursor availability for isoprenoids production, implementation of genetic engineering strategies on host cells is necessary. When the MVA pathway is modified for isoprenoid production, strategies should be implemented to maximize acetyl-CoA accumulation. Deleting or downregulating genes associated with byproduct formation presents a viable option [78]. Down-regulation of the genes aldose reductase (AR), galactose dehydrogenase (GALDH), lactate dehydrogenase (LDH), acetaldehyde dehydrogenase (ADH), pyruvate oxidase (POX), and acetyl-CoA carboxylases (ACCs) can result in more carbon being available for enhanced isoprene synthesis [61].

There are also some reports about introducing the new precursor synthesis pathway/carbon source, e.g., heterologous acetyl-CoA synthetic pathway introduced into *S. cerevisiae* to boost the levels of acetyl-CoA. The dual utilization of mitochondrial and cytoplasmic acetyl-CoA was applied in *S. cerevisiae*, leading to an increase in isoprene production of approximately 2.1- and 1.6-fold, respectively, greater than those strains generated by mitochondrial or cytoplasmic engineering alone [81, 94]. When the MEP pathway is implemented for isoprenoid production, a major limitation is an unequal supply of G3P and pyruvate, as G3P gets converted to pyruvate through multiple steps in glycolysis (Fig. 1). Hence, G3P needs to be balanced toward the biosynthesis of isoprenoids in microalgae. In addition to G3P, pyruvate, and acetyl-CoA in the biosynthesis of isoprenoids, the limited availability of the two main intermediate precursors, IPP and DMAPP generated via MEP or MVA pathway, also diminishes the productivity of the desired isoprenoid products. The synergistic effect of two modifications was investigated to increase isoprene production in *Synechocystis* from 0.2 to 12.3 mg g^−1^. The first modification increased intermediate precursor (DMAPP) availability by overexpressing fni from *Streptococcus pneumonia*, and the second increased cellular IspS protein concentration by expressing IspS as a fusion construct with the cyanobacterial cpcB gene [61]. Eukaryotes, fungi, and some plants function through the MVA pathway in order to produce IPP and DMAPP, while bacteria and cyanobacteria possess the MEP pathway. Unlike these species, diatoms retain both MEP and MVA pathways and convert the obtained precursors to end-products [78]. According to transcriptomic sequencing of numerous species and isotope tracer analysis, it was verified that diatoms generate IPP and DMAPP through both MEP and MVA routes [7, 85]. This causes higher metabolic flux and greater storage of available precursors for the production of isoprenoids [124]. Cvejić et al. [24] presented *P. tricornutum* and *Nitzschia ovalis* produced epibrassicasterol and cholestadienol (sterols) through the MVA pathway by consuming acetate as a carbon source under mixotrophic conditions. Furthermore, they converted the IPP/DMAPP obtained precursors from CO_2_ to phytol via the MEP route [24]. In another study, the human Gult1 gene was expressed in *P. tricornutum* to create a glucose-using heterotrophic transformant. The Embden–Meyerhof–Parnas (EMP) route was frequently embolized, and excessive G6P accumulation inhibits the Gult1 transformant’s heterotrophic development. In the Gult1 transformant, the 6-phosphofructokinase (Pfk), glucose-6-phosphate dehydrogenase (G6pd), and 6-phosphogluconolactonase (Pgl) genes were expressed to boost the EMP and Pentose Phosphate Pathway (PPP) for accelerating G6P metabolic rate. Under heterotrophy, Pfks expressed transformants have 14–33% more biomass than Gult1 transformants and 4 times more biomass than autotrophy. Under heterotrophy, the G6pds and Pgls expressed transformants have 40% more biomass than the Gult1 transformant and 4.5 times more biomass than autotrophy condition [143]. *Skeletonema marinoi* and *Cyclotella cryptica* formed sterols via cyclization of squalene, the first intermediate in the generation of sterols, from mevalonate pathway [41]. *Rhizosolenia setigera* is another diatom that uses MVA pathway in order to produce sterols and HBIs. While*, H. ostrearia* supplies HBIs and β-sitosterol through MEP rout [93].

### Engineering associated enzymes in the biosynthetic pathway of isoprenoids

Constructing a multigene pathway according to the strategies outlined may lead to several enzymes in the MEP and MVA pathways limiting metabolic flux, requiring the optimization of rate-limiting enzymatic conversions. Depending on the engineered strain, these restrictive points may vary. To identify target enzymes for optimization, kinetic flux profiling has been used to identify bottlenecks in an engineered isoprene production strain. After identifying rate-limiting enzymes, various engineering methods have been developed to optimize key enzymes. Screening of enzymes from different species has been utilized to choose the most appropriate enzyme [78].

There are diverse enzymes that might limit the metabolic flux of the MEP and MVA pathways, thereby diminishing precursors supply and finally isoprenoid production. Isopentenyl phosphate kinase (IPK), iso-pentenyl diphosphate isomerase (IDI), 1-deoxy-d-xylulose 5-phosphate synthase (DXS), 1-deoxy-d-xylulose 5-phosphate reductoisomerase (DXR), 2-C-methyl-D-erythritol 2,4-cyclodiphosphate synthase (MDS), 4-hydroxy-3-methylbut-2-en-1-yl diphosphate reductase (HDR) are some of the main rate-limiting enzymes in pathway under various conditions and organisms [7, 10, 23, 78]. Henry et al. [51, 52] represented the regulatory role of IPK and two nudix hydrolases in contributing of isopentenyl phosphate (IP) and IPP production through active phosphorylation/dephosphorylation reactions. In fact, IP to IPP ratio influences the productivity of isoprenoids obtained from MEP and MVA and this indicates interference in the crosstalk mechanism between the two paths. The regulatory roles of these enzymes were observed in several diatoms [6]. One of the outstanding features of most diatoms is that they contain the enzyme IDI, which is integrated into the squalene synthase (SQS), that accelerates the first stage of sterol biosynthesis [28]. In addition, *P. tricornotum*, *R. setigera,* and *H. ostrearia* catalyze the sterol biosynthesis wherein *R. setigera* and *H. ostrearia* FPP synthase and IDI have located in the endoplasmic reticulum (ER) membrane together and this helps the effective conversion of precursors to sterols [28]. In the MEP pathway, the step catalyzed by 1-deoxy-D-xylulose 5-phosphate synthase (DXS) is one of the major limitations. For example, the DXS mRNA expression improved when dark-adapted *P. tricornotum* cells transferred to light and this issue promoted the production of carotenoids through enhancing the precursors flow. Overexpression of DXS in *P. tricornotum* increased the production of fucoxanthin in a 2.4 fold in comparison with the natural strains to with more accumulation of other intermediates such as diadinoxanthin and β-carotene [31]. According to this research, it was confirmed that DXS is a rate-limiting enzyme in diatoms and leads to more metabolic engineering experiments to improve the isoprenoid biosynthesis in *P. tricornotum*. As mentioned previously, most of the diatoms have the enzyme that isomerizes IPP to DMAPP with integration of squalene synthase (IDI-SQS). The reductive dehydroxylation of (E)−4-hydroxy-3-methylbut-2-enyl diphosphate (HMBPP) to IPP and DMAPP is carried out by the enzyme HDR. Diatoms have cytosolic IDI, the enzyme that isomerizes IPP to DMAPP, in a fusion with squalene synthase, as was mentioned earlier (IDI-SQS). While some species have an additional IDI that is oriented at the plastids, other species do not have a second IDI [39]. If these data are accurate, HDR must be an essential component of the plastids’ isoprenoid synthesis, modulating the ratio of IPP to DMAPP for subsequent reactions. Although catalytic residue (F212) at the enzyme’s active site is essential to produce the final product in diatoms, more in-depth research on HDR enzymes from various species is required to determine the precise IPP:DMAPP ratio formed. Based on Shin et al. [115], before the product is released, this residue mediates the proton transfer to the intermediate allyl anion [115]. Species that lack an independent IDI must have developed unique mechanisms for the isomerization of IPP to DMAPP in plastids if this is inadequate to support the synthesis of plastidic isoprenoids. Examples of such mechanisms include the dual targeting of IDI-SQS or the alternative splicing of the IDI-SQS transcript. Another metabolic engineering approach, which is utilized to enhance the production of fucoxanthin and its precursors, is introducing phytoene synthase, a regulator of the metabolic fluxes towards carotenoid synthesis, into the *P. tricornutum* genome [7]. To metabolically engineer cells to produce particular isoprenoids, an ideal IPP:DMAPP ratio must be achieved because the desired molecule depends on this ratio. For instance, sesquiterpenoids or sterols need a 2:1 ratio, but diterpenoids or carotenoids need a 3:1 ratio.

In MVA-containing eukaryotic cells, hydroxy methyl glutaryl reductase (HMGR) is known as the key regulator and rate-limiting enzyme in the early production of sterol and nonsterol isoprenoids, and it is extensively controlled at the transcriptional, translational, and post-translational levels [56]. Jaramillo et al. [56] found that the overexpression of two versions of the native HMGR and a conventional, heterologous squalene epoxidase (SQE) gene in the diatoms *Phaeodactylum tricornutum* resulted in significant differential accumulation of downstream sterol pathway intermediates. Squalene, cycloartenol, and obtusifoliol accumulated in response to HMGR-mVenus overexpression, whereas cycloartenol and obtusifoliol accumulated in response to heterologous NoSQE-mVenus overexpression. Furthermore, all *P. tricornutum* overexpression lines accumulated the end-point sterol 24-methylenecholesta-5,24(24ʹ)-dien-3-ol, and campesterol increased threefold in *P. tricornutum* lines expressing NoSQE-mVenus [56]. HMGR and IDI SQS were two of the genes that were discovered to be continuously increased during the heterologous expression of plant triterpenoid biosynthesis genes in *P. tricornutum* [25]. They are, therefore, excellent candidates for future pathway optimization research.

In a recent study, Fabris et al. [35] investigated the heterologous production of the monoterpenoid geraniol in *P. tricornutum*, which is known for its use in perfumes and insect repellents (Table 2). Despite the presence of a GPP (C10) in this diatom, a precursor of geraniol, it was unable to produce geraniol on its own. To overcome this obstacle, the researchers expressed *Catharanthus roseus* geraniol synthase (CrGES), previously verified as the most effective enzyme for geraniol synthesis in *S. cerevisiae*, into the cytosol of *P. tricornutum* using an episome vector under the control of both an inducible alkaline phosphatase gene promoter (AP1p) and an endogenous constitutive promoter (Phatr3_J492020p). This method ultimately led to high yields of 48.60 μg L^−1^ and 309 μg L^−1^ of geraniol production, respectively, thereby providing proof-of-concept that extrachromosomal expression through plasmids can be used as a useful tool for future metabolic engineering endeavors in *P. tricornutum*.

### Impediment of the competitive pathway

The isoprenoid synthetic pathway in microalgae has implications that influence cell growth, resulting in the formation of alternative and competitive pathways for the synthesis of target isoprenoids. To maximize isoprenoid production, it is necessary to redirect carbon flux away from these bypass and competitive pathways. However, when the bypass pathway is essential for cell viability, complete knockout of the enzyme can be lethal [125, 129, 137]. Hence, for a successful engineering plan utilizing carbon redirection the physiological effects on cell growth and reproduction must be taken into account by analyzing a variety of output metrics. Comprehending the functioning of homeostasis in engineered cells necessitates a thorough understanding of central carbon metabolism. To address this issue, downregulation strategies at the transcriptional level have been proposed including the selection of a weaker promoter or the use of RNA interference technology [78]. For sesquiterpenoid production from FPP, squalene production from FPP catalyzed by SQS (ERG9) is considered a bypass pathway that needs to be inhibited. Wichmann et al. [132] managed to produce (E)-α-bisabolene, a sesquiterpene biodiesel precursor, in the *C. reinhardtii* cytosol. This was accomplished through threefold overexpression of *Abies grandis* bisabolene synthase (AgBS), which converts FPP to (E)-α-bisabolene, as well as knockdown of the ADP-glucose pyrophosphorylase small subunit (AGPP), farnesyltransferase (PFT), and squalene synthase (SQS). Through this strategy, the engineered strain achieved a 15-fold increase in titer at 10.3 ± 0.7 mg g^−1^ DCW as compared to single expression of AgBS. Furthermore, under light/dark cycle conditions, the titer further increased to 11 mg L^−1^ due to the increased availability of FPP for ubiquinone synthesis in the mitochondria during dark cultivation for respiration. Similarly, Kajikawa et al. [59] attempted to overexpress endogenous squalene synthase and downregulate squalene epoxidase in order to increase squalene production.

### Introducing novel pathway for accumulating precursors

The engineering strategies outlined above are implemented using the native MEP and MVA pathways. To minimize the metabolic load associated with multi-gene expression and the buildup of toxic intermediates, new pathways have been developed. Multiple studies leveraging the novel MVA pathway to generate isoprene have been reported in the literature, wherein fatty acid decarboxylase and oleate hydratase were incorporated to create isoprene from mevalonate in two steps [136]. In novel pathway I, mevalonate-5-pyrophosphate decarboxylase (MDD) catalyzes the isoprenol production reaction from mevalonate. In novel pathway II, MDD catalyzes mevalonate-5-phosphate (MVAP) to produce IP, and phosphatase subsequently catalyzes a reaction which produces isoprenol. The low activity and affinity of fatty acid decarboxylase, oleate hydratase, and MDD resulted in a decreased production of isoprene or isoprenol. Hence, further studies to identify enzymes with better activity and higher affinity are essential. In comparison to the novel MVA pathway, there has been limited research on the novel MEP pathway. Hence, the lack of efficient enzymes in these pathway continues to hinder its application, necessitating enzyme screening and engineering to improve metabolic flux [44].

Novel engineered pathway for hemiterpenoid production has been largely confined to the production of isoprene and isopentenol, simple five-carbon molecules that do not involve IPP/DMAPP condensation [78]. Consequently, examining a novel pathway which eliminates the long pathway but does not bypass IPP and DMAPP synthesis would be more valuable and could be applied to all types of isoprenoid production. In two studies, a two-step pathway for IPP and DMAPP generation from prenol and isoprenol was discovered. Two kinases were identified that catalyze the transformation of prenol and isoprenol to DMAP and IP, respectively. This separate metabolic route from the main carbon metabolism has potential for high metabolic flux, with Stephanopoulos'group's pathway showing adequate metabolic flux for nearly all downstream pathways tested. Nonetheless, further research is needed to supply inexpensive and sustainable supplies of prenol and isoprenol substrates in order to enable utilization of this novel pathway for additional isoprenoid synthesis [15, 88].

In addition to the native pathway, another novel pathway for the synthesis of 1-deoxy-d-xylulose-5-phosphate (DXP) of the MEP pathway from ribulose 5-phosphate (Ru5P) through a reaction catalyzed by YajO, a putative xylose reductase, and mutant RibB was investigated by Kirby et al. [68]. This novel pathway does not rely on the central carbon system and bypasses the rate-limiting step, which catalyzes the conversion of G3P and pyruvate to DXP. Consequently, it is promising for high metabolic flux towards the isoprenoid pathway without diminishing normal cell growth [68]. Thus, precise control and regulation of biosynthetic pathways have been made possible by the employment of synthetic biology technologies, such as dynamic gene circuits and synthetic promoters, particularly in the synthesis of high-value isoprenoids. In this regard, there is notable research by Einhaus et al. [33], which focused on rational promoter engineering in *C. reinhardtii*. They created novel synthetic expression elements by systematically evaluating existing expression elements and integrating them with rational promoter engineering. This approach revealed a synergy between the PSAD 5′ untranslated region (UTR) and its corresponding chloroplast targeting peptide, and it also enhanced the uniform application of synthetic biology techniques. The developed synthetic algal promoter enhanced the production of the sesquiterpene (E)-α-bisabolene by four times when compared to commonly used expression elements and eighteen times when compared to its native form [33].

These techniques greatly increase yield and stability by enabling real-time adaptation to metabolic or environmental signals. Algal biotechnology is undergoing a paradigm shift as a result of these developments, shifting toward more reliable, scalable, and effective engineering techniques.

In the end, proper selection of a microalgal host for biotechnological applications critically depends on its isoprenoid biosynthesis pathways. Microalgae such as diatoms possessing both MVA and MEP pathways offer distinct advantages over MEP-only species. For the synthesis of high-value isoprenoids, such as terpenoids, carotenoids, and sterols, the dual-pathway architecture offers improved precursor availability and compartmentalized flux distribution, resulting in increased metabolic flexibility [6]. In addition, when compared with the plastid-confined MEP system, the cytosolic location of the MVA pathway enables more accessible and effective metabolic engineering approaches. On the other hand, the MEP pathway is preferred for CO_2_-based isoprenoids production because it is stoichiometrically more efficient, requiring just six molecules of CO_2_ for the synthesis of either IPP or DMAPP, in comparison to the nine molecules needed for the MVA [32]. These advantages must be balanced, though, with the technical drawbacks of diatoms, which include complex regulatory systems and less advanced genetic tools than those of model green algae [18]. Nevertheless, microalgae with the MVA route have greater potential for producing isoprenoids, their efficient use necessitates ongoing developments in systems biology and genetic engineering technologies. The precise biosynthetic objectives and the organism’s engineering capabilities should therefore serve as guidance when choosing the best host.

## Emerging metabolic engineering tools

In the last 10 years, significant progress has been achieved in expanding the range of tools available for genetically modifying microalgae, increasing their potential as model organisms for use in both industrial and scientific applications. These include Zinc-Finger Nucleases (ZFNs), Transcription Activator-Like Effector Nucleases (TALENs), and (CRISPR/Cas) tools [1, 2]. These techniques mostly cause DNA double-strand breaks, which in turn initiate the cell’s natural DNA repair mechanisms. By applying this method, donor DNA can be added for transgenic integration, or targeted genes can be disabled.

ZFNs are artificial proteins that bind DNA and have two domains connected by a linker sequence. Through a zinc-finger DNA binding domain and a restriction endonuclease FokI-mediated DNA cleaving domain, they offer sequence specificity [5]. However, because of their complexity, difficulties, and expense, their application has been restricted, leading scientists to create a more straightforward, efficient, and economical technique for precise genome modification.

TALENs are specially designed tools that merge DNA-binding domains from TALE proteins with the FokI cleavage domain. Each repeat domain of these transcription activator-like effectors (TALE) proteins can recognize a single base pair. In order to allow precise genome editing, TALENs induce specific double-strand breaks (DSBs) in DNA, which initiate repair processes. The TALENs technology functions essentially as a specially designed restriction enzyme that can target particular genomic sites. Meganucleases and TALE nucleases were both employed in a recent study to change the diatom *P. tricornutum*. The transformed strain produced more oil as a result of their effective editing of seven genes necessary for lipid synthesis [26, 40, 71]. This highlights how TALENs can be used to precisely alter genes and improve desirable characteristics in microalgae.

Although all of these genetic modification tools have created new opportunities for the bioproduction of economically significant compounds using microalgae, CRISPR is the most promising technology for the future and is preferred in microalgae genetic engineering. Scientists are able to identify the roles of several genes in metabolism, growth, and other cellular processes of microalgae by employing CRISPR technology, which allows for specific gene modification or knockout, specifically in diatoms like *P. tricornutum*. Furthermore, CRISPR-based editing has been utilized to produce microalgal strains with superior characteristics for a range of applications, including higher biomass productivity, improved stress tolerance, and increased lipid and pigment production. Despite some challenges, the CRISPR/Cas technique has been effectively used for developing microalgal strains for several kinds of applications in industry. In biofuel production, researchers enhanced overall lipid productivity in *C. reinhardtii* by 64.25% without noticeably altering cell growth when they knocked out the phospholipase A2 gene, leading to a greater diacylglycerol pool and increased triacylglycerol accumulation [123]. In another study, the genes involved in acyl-CoA use, including fatty acid synthesis, β-oxidation, and autophagy, were impacted by functional knockout of the acyl-CoA binding protein (ACBP), especially when cells were dependent on endogenous carbon deposits. Triacylglycerols (TAGs) lipolysis was also inhibited by knocking out this gene, and this was linked to the inhibition of lipid droplets/TAGs degradation observed in previous studies [76]. CRISPR is also used for enhancing photosynthetic efficiency and pigment production. Recently, the CpSRP54 gene, which is essential for *P. tricornutum*’s indirect carbon fixation pathways, has been targeted using a CRISPR-based strategy. This alteration highlights the potential of CRISPR/Cas9 to fine-tune metabolic pathways that are essential for optimizing carbon utilization and storage, hence increasing the organism’s applicability in industrial applications [75]. However, industrial applications of microalgae need to achieve the same growth rate as bacteria and yeast. One significant development is the one-step transformation of *C. reinhardtii* using a DNA-free CRISPR/Cas9 technique, which allowed the CpFTSY and ZEP genes to be sequentially knocked out. In dense cultures, light penetration was improved by the reduction in chlorophyll antenna size caused by the CpFTSY gene deletion. Moreover, a strain with a markedly elevated zeaxanthin production without compromising photosynthesis was produced by altering the ZEP gene. This method is crucial for optimizing biomass production in systems of large-scale cultivation. Targeting the photosynthetic system in microalgae not only improves light usage but also increases pigment yields for commercial applications. Since there are many pigments associated with the photosynthetic system, such as carotenoids, astaxanthin, lutein, phycoerythrin, and phycobiliproteins, which are useful for human consumption [57].

## Techno-economic and life-cycle assessment of microalgae-based isoprenoids

Techno-economic analysis (TEA) and life-cycle assessment (LCA) are essential tools for the evaluation of the industrial viability of microalgae as a platform for isoprenoid production. Cost-effectiveness of the recent TEAs involves several factors such as biomass productivity, downstream processing efficiency, capital investment, and operating expenses. Based on the recent findings, it suggests that while microalgae-based systems are currently more expensive than conventional microbial platforms, their costs can be significantly reduced through advances in metabolic engineering strategies and process integration for co-product recovery (e.g., lipids or proteins) as mentioned earlier [65, 116]. In parallel, LCAs evaluate the environmental sustainability of microalgal isoprenoid production, accounting for energy input, greenhouse gas emissions, water use, and land footprint. Several LCA studies have demonstrated that microalgal production systems, particularly those using CO₂ capture and wastewater as inputs, offer lower environmental impacts compared to fossil-based or sugar-fed microbial processes. However, the energy-intensive nature of harvesting and drying algae remains a key environmental hotspot. Integrating renewable energy sources and optimizing bioprocesses via engineered strains can further enhance the sustainability profile. Together, TEA and LCA highlight that while microalgae-based isoprenoid production is not yet cost-competitive at commercial scale, it holds strong long-term potential given its renewability, co-product synergy, and carbon neutrality [79].

## Conclusions and future perspectives

Microalgae have the potential to be employed as a sustainable source of biochemical and bioactive compounds using organic and inorganic carbon sources. The recent advancements in genetic engineering have enabled the engineering of microalgal strains to increase both biomass productivity and the yield of high-value products. Various studies have evaluated the metabolic engineering strategies adopted to optimize the MEP and MVA pathways for isoprenoid production in microalgae. This review summarizes findings published in the recent five years. Engineering of isoprenoid biosynthesis in heterologous hosts offers the potential to produce adequate quantities and various structures of these compounds to make them industrially and commercially applicable. Despite considerable progress, microalgal metabolic engineering has encountered various challenges. To date, random integration has been the primary method of microalgal genetic engineering as episomal plasmid expression and homologous recombination are not available to most microalgae. As a result, transgene integration sites have led to unexpected and inconsistent phenotypes. In addition, unstable expression and silencing of transgenes have impeded the development of microalgae metabolic engineering. Fortunately, innovative gene editing tools such as RNAi, CRISPR/Cas9, ZNFs, and TALENs or intron-mediated gene expression are promising for producing more sophisticated and stable transformants in the future [103, 119]. Besides, advancements in metabolic engineering of microalgae could be facilitated by gaining a comprehensive understanding of the location and purpose of specific metabolic pathways, as well as having the ability to manipulate them. However, compared to simple organisms like bacteria, it remains a challenge to manipulate the genome of microalgae. Finally, difficulties arise when attempting to find low-cost and eco-friendly techniques for extracting all bioactive components from microalgal cells. Furthermore, some economic and environmental constraints hinder large-scale applications.

Implementation of metabolic engineering approaches in microalgae offers novel platforms for the generation of terpenoids requiring less expensive growth conditions and possessing a background metabolism that may be more suitable in comparison to the existing microbial production platforms. Despite the considerable progress made in the past decade in elucidating the biosynthesis of isoprenoids within the microalgal group, there are still certain biosynthetic pathways that have to be investigated. Exploring these unknown pathways could further enhance our comprehension of the entire process and distinguish between microalgae-associated products.

## Data Availability

No datasets were generated or analysed during the current study.
